# A loop-mediated isothermal amplification (LAMP) assay to identify isotype 1 β-tubulin locus SNPs in synthetic double-stranded *Haemonchus contortus* DNA

**DOI:** 10.1007/s12639-021-01414-w

**Published:** 2021-07-05

**Authors:** Livio M. Costa-Junior, Umer N. Chaudhry, Philip J. Skuce, Seamus Stack, Neil D. Sargison

**Affiliations:** 1 Pathology Department, Federal University of Maranhão, São Luís, Maranhão Brazil; 2 Royal (Dick) School of Veterinary Studies, University of Edinburgh, Roslin, Midlothian, EH25 9RG UK; 3 Moredun Research Institute, Pentlands Science Park, Edinburgh, Midlothian, EH26 0PZ Scotland, UK; 4 Mast Group, Mast House, Derby Road, Bootle Merseyside, L20 1EA UK

**Keywords:** Benzimidazole, Resistance, Nematode, Small ruminant, LAMP

## Abstract

Development of sustainable gastrointestinal nematode (GIN) control strategies depends on the ability to identify the frequencies of drug-susceptible and resistant genotypes in GIN populations arising from management practices undertaken on individual farms. Resistance to BZ drugs in GINs has been shown to be conferred by the presence of defined SNPs in the isotype 1 β-tubulin locus. Loop-mediated isothermal amplification (LAMP) assays are amenable to use on a range of DNA templates and are potentially adaptable to use in practical, cost-effective, pen-side diagnostic platforms that are needed to detect anthelmintic resistance in the field. In this study, we designed primers and examined LAMP assays to detect each of the three major isotype 1 β-tubulin SNPs conferring genetic susceptibility to BZ drugs. We used artificial pools of synthetic DNA, containing different proportions of susceptible and resistant SNPs to determine reproducibility of the assays. We demonstrated the detection of each of the isotype 1 β-tubulin SNPs conferring susceptibility to BZ drugs using the optimal LAMP assay. Isotype 1 β-tubulin SNP typing was effective in detecting BZ susceptibility, but the accuracy was reduced in samples with less than 60 % susceptible DNA. Our results show the potential for LAMP SNP typing to detect genetic susceptibility or resistance to anthelmintic drugs in livestock GINs, and some of the limitations in our approach that will need to be overcome in order to evaluate this assay using field samples.

**Supplementary Information:**

The online version contains supplementary material available at 10.1007/s12639-021-01414-w.

## Introduction

Gastrointestinal nematodes (GINs) are a major global cause of production loss in livestock (Nieuwohf and Bishop 2005; Lane et al. 2015; Charlier et al. 2020). Drenching with synthetic anthelmintics is the main form of control worldwide, but resistance has been reported to each of the single active broad-spectrum anthelmintic drugs that are available for use in small ruminants, with little foreseeable prospect for the development of a new single-active, broad-spectrum anthelmintic drug group (Kotze and Prichard 2016; Mazzucato 2016). The benzimidazole (BZ) drugs have been widely used in veterinary and human medicine since their launch in the early 1960 s (Lacey 1990), but resistance is commonplace in small ruminant GIN populations around the world (Bartley et al. 2003; McKenna 2010; Veríssimo et al. 2012; McMahon et al. 2013; Playford et al. 2014; Keegan et al. 2017). These drugs must therefore be used responsibly (Kaplan and Vidyashankar 2012).

Anthelmintic resistance can be detected phenotypically in the field using the faecal egg count reduction test (FECRT), or *in vitro* using egg hatch (EHT) or larval development tests (Taylor et al. 2002). However, these methods can be laborious, require technical expertise, and may be insensitive at low levels of resistance (Coles et al. 2006; von Samson-Himmelstjerna et al. 2009a; Torres-Acosta et al. 2012). Molecular methods to detect the genetic changes underlying anthelmintic resistance and platforms for their deployment in the field are, therefore, needed to help in the development of strategies for the responsible and sustainable use of BZ drugs (Nunes et al. 2013).

The primary mode of action of BZ drugs involves their interaction with the cytoskeletal protein, β-tubulin (Lacey 1990). BZ resistance in GINs has been shown to arise due to mutations in the isotype 1 β-tubulin locus (Kwa et al. 1993). Genotypic tests for BZ resistance are based on the detection of key mutations [F200Y (TAC), F167Y (TAC) and E198A (GCA) or E198L (TTA)] at this locus. Various PCR assays have all been described for BZ resistance in a variety of species (Ye et al. 2001; Alvarez-Sánchez et al. 2005; Tiwari et al. 2006; Diawara et al. 2009); and pyrosequencing (von Samson-Himmelstjerna et al. 2009b; Chaudhry et al. 2015) and deep amplicon sequencing (Avramenko et al. 2019; Sargison et al. 2019) platforms have been developed to detect low frequencies of resistance mutations in parasite populations (Roos et al. 1995; Diawara et al. 2009; Avramenko et al. 2019). However, these platforms can be relatively expensive and slow in reporting results for field application. An important challenge is, therefore, to develop molecular tests that can be routinely used in the field to assess the frequencies of resistant genotypes in parasite populations (von Samson-Himmelstjerna 2006).

An alternative DNA amplification technique to PCR, known as loop-mediated isothermal amplification (LAMP) has been developed (Notomi et al. 2000). The LAMP reaction uses two sets of primers, outer primers (F3 and B3) and inner primers (FIP and BIP) that hybridize to six sites on the target DNA. This generates a mixture of stem-loops containing inverted repeats and results in exponential amplification of the target sequence (Poole et al. 2017). A key distinction is that LAMP is performed under isothermal conditions, using *Bst* polymerase rather than *Taq* polymerase, requiring relatively simple equipment and reagents and is, therefore, adaptable to use at the point-of-care, and/or under field conditions (Poole et al. 2017; Rodriguez-Garcia et al. 2018).

LAMP offers a valuable platform for nucleic acid detection (Notomi et al. 2000) under field conditions, using different source samples, with robust results, low cost and minimal skill requirements (Liu et al. 2018; Yang et al. 2018). This method has been largely used to diagnose viral, bacterial and protozoal infections, and more recently, helminth parasites of humans and animals Notomi et al. 2000; Obura et al. 2011; Shiraho et al. 2016; Rodriguez-Garcia et al. 2018; Srividya et al. 2019), including *Haemonchus contortus* Iseki et al. 2007; Melville et al. 2014; Lopes-Jimena et al. 2018; Wu et al. 2018; Deng et al. 2019).

Most of the aforementioned LAMP applications determine the presence or absence of pathogen DNA, or its relative abundance. The use of LAMP for SNP analysis is more complex, relying on the principle that if the SNP is present, the DNA structure will be altered and amplification will occur, but if it is not, the DNA structure will not change and amplification cannot occur. LAMP assays have been described for SNP genotyping of different cell types (Kumasaka et al. 2016; Kwong et al. 2018; Matsumoto et al. 2018; Yamanaka et al. 2018; Du et al. 2019), and to detect fungicide and herbicide resistance in weeds (Pan et al. 2015; Fan et al. 2018). LAMP assays have also been described to detect isotype-1 β-tubulin SNPs conferring BZ resistance in human soil-transmitted helminths, *Trichuris trichiura*, *Necator americanus* and *Ascaris lumbricoides* (Rashwan et al. 2016, 2017); and to detect a rare E198A (GCA) isotype-1 β-tubulin SNP in *Haemonchus contortus* (Tuersong et al. 2020).

The development of a SNP genotyping LAMP method that could be used as a pen-side test to detect isotype-1 β-tubulin SNPs in field populations of GINs would be useful to support responsible anthelmintic resistance mitigation on individual farms. The aim of the present study was to investigate the feasibility of a LAMP assay to detect three known isotype-1 β-tubulin SNPs conferring BZ resistance, using synthetic double-stranded DNA fragments of the important abomasal blood-feeding GIN parasite of ruminant livestock, *H. contortus*.

## Materials and methods

### LAMP primer design

Isotype-1 β-tubulin sequences of *Teladorsagia circumcincta*, *Trichostrongylus colubriformis*, *Oesophagostomum columbianum* and *H. contortus* were downloaded from GenBank and consensus sequences were compiled using CLUSTAL W of Jalview 2.10.5 (Waterhouse et al. 2009) (Supplementary material S1). This allowed the design of LAMP primers specific to *H. contortus* that would not cross-react with the DNA of the other species. Degenerate consensus sequences were then created to allow all genotypes to be amplified using IUPAC (International Union of Pure and Applied Chemistry) code by Bioedit software (Hall 1999). The F200Y (T**T**C to T**A**C), F167Y (T**T**C to T**A**C), and E198A (G**A**A to G**C**A) isotype-1 β-tubulin SNPs were identified and forward and reverse outer LAMP primers (F3 and B3) designed, as well as forward and reverse inner LAMP primers (FIP: F1c-F2 and BIP: B1c-B2, respectively), using the online primer designing software (Primer Explorer v.4: Eiken Chemical, Japan) (Table 1). The FIP and BIP primers consisted of parts of the sequence that were complementary to the forward strand (F2 or B1c) and to the reverse strand (F1c or B2), becoming a loop in the amplification. The primers were designed to amplify the susceptible genotype (to discriminate between susceptible and resistant genotypes with at least seven minutes of difference) using the SNP at the end of BIP (B2) for F167Y (T**T**C); at the beginning of FIP (F1c) for E198A (G**A**A); and at the beginning of BIP (B1c) for F200Y (T**T**C) (Table 1). All LAMP primer sequences were degenerated using IUPAC code and were submitted to BLASTn to *in silico* confirm that they would amplify the *H. contortus* isotype-1 β-tubulin locus.

**Table 1:** LAMP primers for isotype 1 β-tubulin SNP detection in *Haemonchus contortus*

SNPs - Primer	Sequences 5′ − 3′
SNP 167	
S167F3-285-54	GCTTCAACTYTDATGDGTGA
S167B3-285-54	GHTTDNCACGATCTCACCTTG
S167FIP-285-54	ATCCAGTGCCTCCTCCAAGTATAHATTTCAAHTYGTRCTCAG
S167BIP-285	CTGGAATGGGCACTTTGTAAATTTCGTGATGGAACAACGGAGA
SNP 198	
d198F3-149	GGAADATGTTTTAAGGTATCCG
198B3-149	ACCAAGGTGGTTGAGATC
dS198FIP-149	TCATCGGTGTTYTCTACCARTTACTGTYGTVGAACCCTACA
198BIP-149*	AACAT**H**CTGTATTGACAACGAAGCTATAGGTTGGATTTGTGAGTTTC
SNP 200	
200F3-150	TGTTTTAAGGTATCCGACACT
d200B3-150	RGYHTADGTATACTHTDGBAAGKGT
d200FIP-150	TGTTDCATCGGTGTTYTCTACCAGTYGTVGAACCCTACAATGC
S200BIP-150	TTCTGTATTGACAACGAAGCTCTGGGTTGAGATCTCCATAGGTT

The underlined nucleotides show the SNP. * The bold nucleotide show the SNP 200 position. The primers were degenerate using IUPAC code. Y: C/T; K: G/T; R: A/G; D: A/G/T; H: A/C/T; V: A/C/G; B: C/G/T; N: A/C/G/T. All of the synthesised primers were HPLC grade (Integrated DNA Technologies, Leuven, Belgium)

### Optimisation of the LAMP assay

699 bp double-stranded DNA fragments of susceptible and each of the three resistant isotype-1 β-tubulin from *H. contortus* were synthesised with high-fidelity synthesis chemistries (gBlocks® Gene Fragments, Integrated DNA Technologies) and used as DNA template to optimise the LAMP assay. The reactions were performed using the MAST ISOPLEX®DNA Lyo amplification kit (Mast Group, Bootle, UK, product code DNA/LYO1). Kit reagents were resuspended in a mix containing 10 µl Tris reconstitution buffer (0.01 or 0.1 M), FIP and BIP (0.8, 1.6, or 3.2 µM) primers, F3 and B3 (0.2, 0.4, or 0.8 µM) primers, and the presence or absence of bovine serum albumin (BSA) (6.02 mM). A first round of analytical sensitivity was performed in duplicate using 0.1, 0.05, 0.01, 0.005 ng/µl of susceptible DNA of isotype-1 β-tubulin from *H. contortus*. The reactions using different primer concentrations were incubated for 60 min in a Rotor-Gene Q system qPCR device (Qiagen, Hilden, Germany) at different temperatures (63, 65 and 67 °C). Fluorescence was measured each minute (while not technically referring to a cycle, this was considered in the machine as a Ct value in order to standardise the results) with the temperature staying constant, and the threshold values were obtained with the baseline of template blank controls. The results were expressed in minutes. Template blanks were used in all of the LAMP reactions as a negative control. Reactions with blank amplification in 60 min were not used.

### Diagnostic value of the LAMP assay

The susceptible and one of the three resistant fragments of the *H. contortus* isotype-1 β-tubulin DNA were mixed in artificial pools with 100, 90, 80, 60, 40, 20 and 0 % of susceptible DNA. The LAMP reactions were performed in triplicate using 0.005 ng of DNA from artificial pools under the best conditions selected from the optimisation steps. The results in minutes (referred to here as Ct) were used to generate equations by linear regression using GraphPad Prism 7.0 software (GraphPad Inc., San Diego, CA, USA). These were used to generate a Ct cut-off for detection of 40, 60, 80 and 90 % susceptible DNA by concentration.

Samples (35 for SNP 167, 17 for SNP 198, and 37 for SNP 200) with different DNA concentrations (0.005, 0.01, 0.05, 0.01 and 0.05 ng, respectively) were used to calculate the minimum frequency at which a susceptible or resistant allele could be defined relative to the template blank control (loosely referred to as sensitivity) and the proportion of resistant samples that were correctly identified (loosely referred to as specificity) using GraphPad Prism 7.0 software (GraphPad Inc.). This software calculates these values using each value in the data table as the cut-off value.

## Results

### Optimisation of LAMP conditions

Sets of primers (F3, B3, FIP and BIP) were designed to amplify isotype 1 β-tubulin alleles [F167Y (TTC), E198A (GAA), F200Y (TTC)] conferring susceptibility in *H. contortus* to BZ drugs. Screening of these primer combinations was performed using a first round with different concentrations of FIP and BIP (0.8, 1.6, or 3.2 µM) primers, and F3 and B3 (0.2, 0.4, or 0.8 µM), without BSA at temperature of 67 °C for 60 min.

The sets of primers were selected that showed increased fluorescence without the amplification of blank template controls in under 50 min (Ct), and a difference between the amplification of susceptible and resistant isotype 1β-tubulin SNPs that was longer than seven minutes (Ct) to allow for the possibility of transfer to a colorimetric assay in future stages of development. A total of five sets of primers was designed to amplify susceptible F167Y isotype 1 β-tubulin alleles, from which one was selected (rejected primer sets are shown in Supplementary material S2). The first sets of primers designed to amplify susceptible E198A, and F200Y β-tubulin alleles were selected. The selected primers are described in the Table 1.

**Fig. 1:**
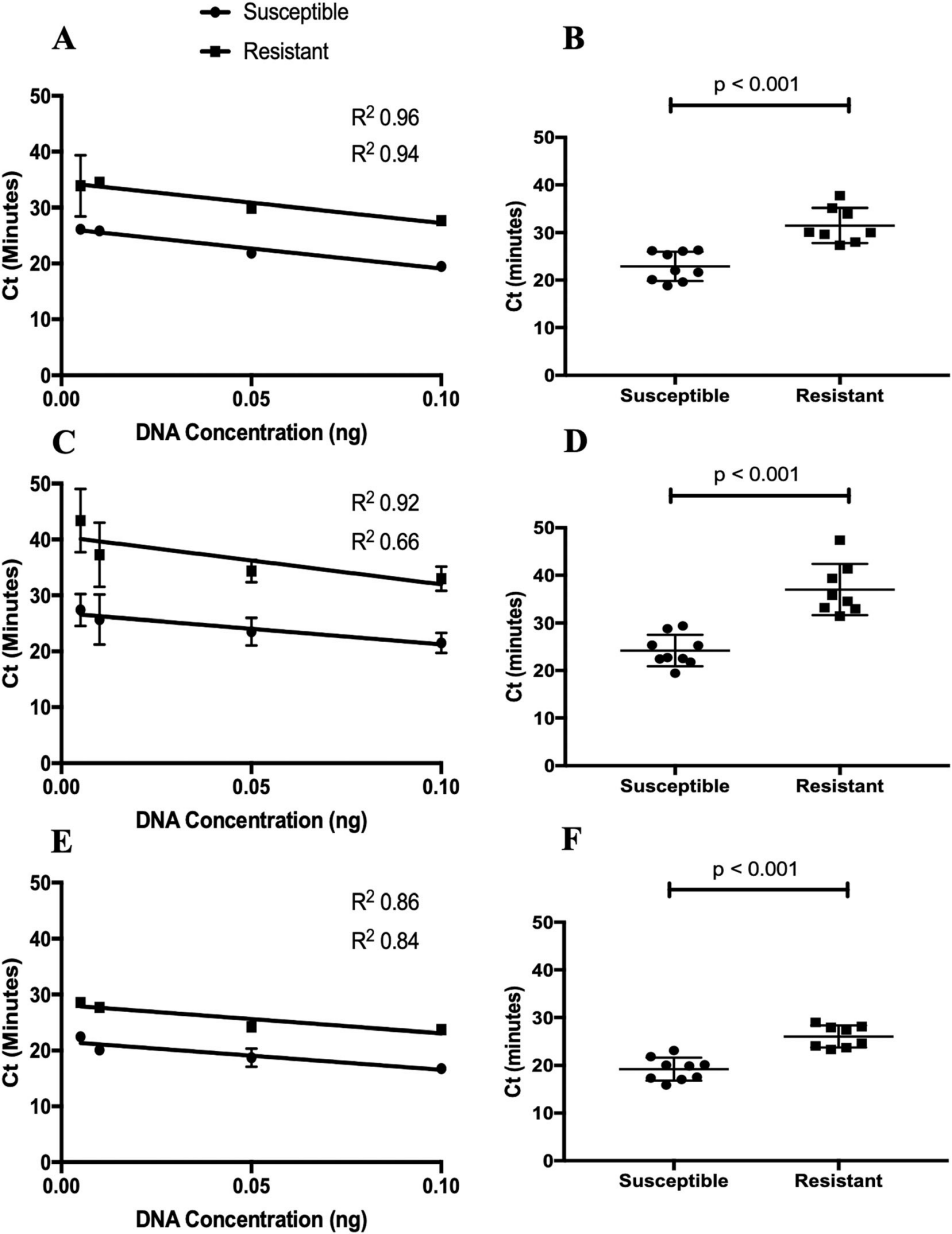
Time (Ct minutes) to detect fluorescence by LAMP using different concentrations of DNA template representing susceptible and resistant *Haemonchus contortus* isotype 1 β-tubulin SNPs in biological replicates at codon 167 (**a** and **b**), codon 198 (**c** and **d**) and codon 200 (**e **and **f**). Fluorescence was measured each minute (considered in the machine as a cycle - Ct)

The optimal LAMP assay conditions were at an incubation temperature of 67 °C for 60 min. The best primer concentrations to discriminate between susceptible and resistant genotypes with different concentrations of DNA template were 3.2 µM for FIP and BIP and 0.8 µM for F3 and B3, along with 0.01 M Tris buffer and 6.02 mM BSA (Fig. 1).

### Sensitivity and specificity

**Fig. 2:**
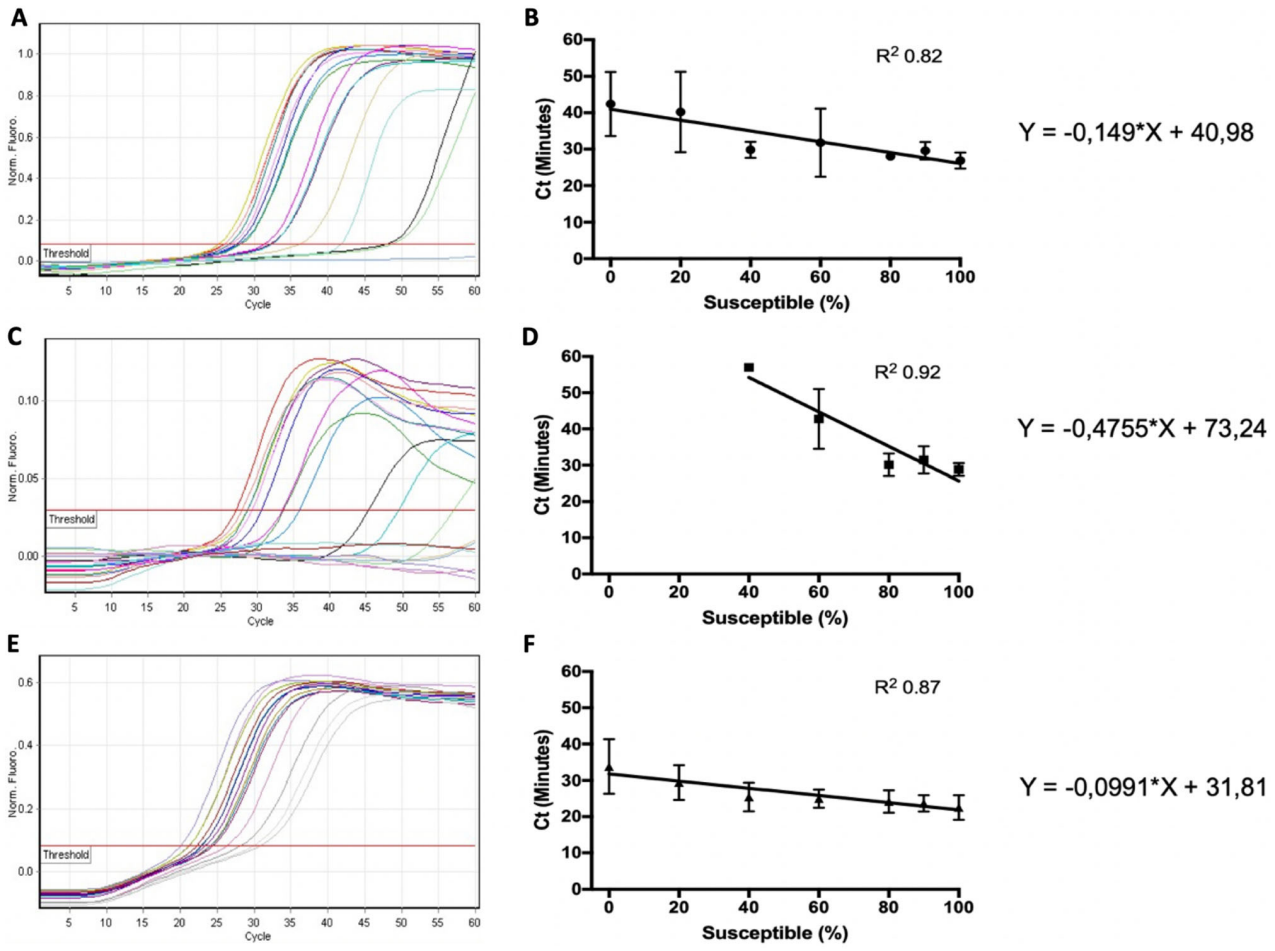
Representative fluorescence of the respective LAMP assays with different concentrations of BZ susceptible *Haemonchus contortus* DNA with F167Y (**a** and **b**), E198A (**c** and **d**) and F200Y (**e** and **f**) SNPs. Fluorescence was measured at each minute (considered in the machine as cycle - Ct). The Ct (minutes) when the fluorescence passed the threshold was used as result. The equations were obtained from linear regression using as independent variable the concentration of susceptible DNA and dependent variable the results in Ct (min)

**Fig. 3:**
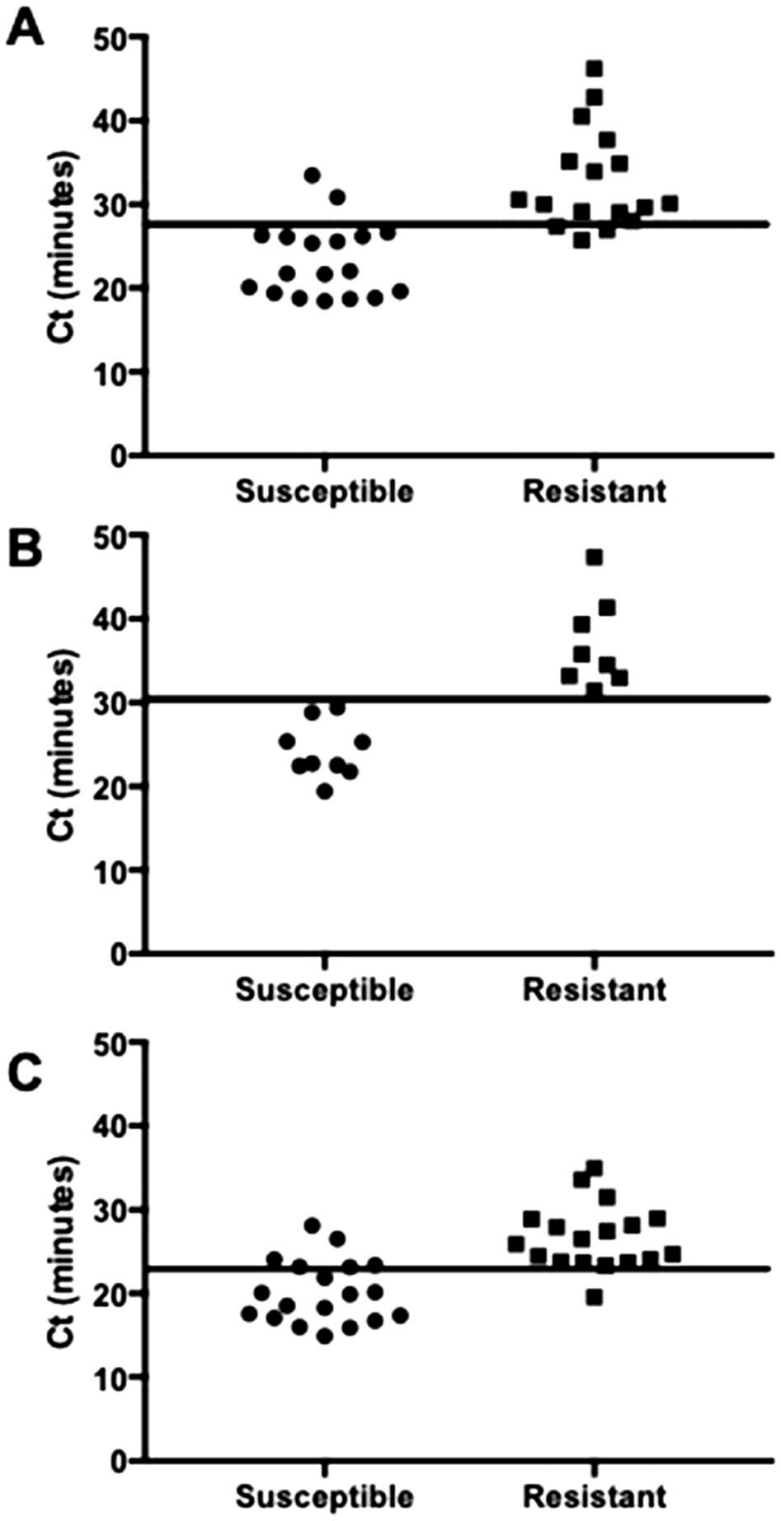
Differences in Ct (min) amplified by LAMP between susceptible and resistant mutations in codon 167 (**a**), 198 (**b**) and 200 (**c**) of *Haemonchus contortus*, with a cut off to detect 90 % (dashed line) of susceptible. Cut off values were calculated using the linear regression described in Fig. 2

The sensitivity and specificity of the optimised LAMP assays to detect BZ susceptible isotype-1 β-tubulin SNPs were calculated using artificial pools with different percentages of BZ susceptible DNA. Linear regression was performed to generate equations to describe the relationship between Ct values and the proportions of the respective F167Y, E198A, and F200Y isotype 1 β-tubulin SNPs (Fig. 2). These equations were used to calculate the cut-off Ct values to detect 90 % of susceptible SNPs in each artificial pool. These were 27.6, 30.4, and 22.9 min of reaction for F167Y, E198A, and F200Y, respectively (Fig. 3). These cut-off values were used to calculate the sensitivity and specificity of the LAMP assays to detect 90 % of susceptible SNPs. The sensitivities of LAMP assays were highest for F167Y (89 %) and E198A (100 %), respectively and lowest for F200Y (68 %). The specificity values were high, being 82 %, 100 and 94 % for F167Y, E198A, and F200Y, respectively.

## Discussion

In the present study, we designed and tested different sets of primers to detect three SNPs conferring susceptibility or resistance to BZ drugs in *H. contortus*. Degenerate primer sets were designed using IUPAC code due to the high level of genetic variability in the *H. contortus* isotype-1 β-tubulin locus (Beech et al. 1994). This strategy increased the possibility of amplification of different genotypes (Marmesat et al. 2016; Lol et al. 2019). The reaction temperature of 67 °C and a low concentration of Tris buffer allowed the amplification to be more specific in identifying susceptible SNPs when using the degenerate primers (Lebedev et al. 2008). This increased the ability of the LAMP assay to discriminate between BZ susceptiblility and resistance with significant differences between the times (Ct – minutes) of amplification (*p* < 0.001), even with low concentrations of DNA template.

We optimised our LAMP reaction using fluorescence detection in a qPCR device to identify the BZ susceptible isotype-1 β-tubulin genotypes. One advantage of LAMP over conventional molecular techniques is that sophisticated equipment is not required for confirming test results and the product could potentially be visualised: either with the naked eye (Shiraho et al. 2016); using a smartphone-based diagnostic platform (Ganguli et al. 2017; Priye et al. 2017); or using electrochemical sensors (Safavieh et al. 2014). Further validation is needed before these platforms can be deployed under field conditions (Kreutz et al. 2019).

The LAMP assays showed high repeatability and sensitivity for detecting BZ resistance or susceptibility in artificial pools containing more than 80 % of susceptible synthetic DNA within Ct reaction times of 15–30 min, in a single amplification and detection step. Other molecular tests for BZ resistance SNPs are potentially more sensitive and specific in detecting different frequencies of resistance mutations in parasite populations (Alvarez-Sánchez et al. 2005; Tiwari et al. 2006; von Samson-Himmelstjerna et al. 2009b; Avramenko et al. 2015, 2019; Baltrusis et al. 2018) for use in experimental studies and research. However, the main advantage of the LAMP is for use in field conditions to detect early stages of resistance and help to reduce further selection, or inform mitigation strategies. The sensitivity of the LAMP assays was low for F200Y (68 %), which might limit its use in the epidemiological studies; but may be adequate when selecting appropriate drugs to treat animals in field conditions.

We have described the development of a LAMP assay using synthetic DNA and described its repeatability in the detection of susceptibility to BZ drugs conferred by three SNPs in the isotype-1 β-tubulin locus. We adopted this approach using synthetic DNA rather than genomic DNA derived from experimental or field samples as our approach to control variation and best understand the assay conditions and variables. Although we identified significant differences between susceptible and resistant templates, the approach may be less feasible when applied to field samples yielding poorer quality or more polymorphic DNA. We, therefore, acknowledge our results as being preliminary and that further work is needed using genomic DNA templates containing heterozygous genotypes and derived from field samples. Nevertheless, our results show the potential to further refine this assay: to improve its sensitivity; to apply it to genomic DNA to detect at the same time the three isotype-1 β-tubulin SNPs associated with BZ resistance in field samples, and to develop point-of-care platforms for its use. One issue to address may be the use of time to threshold (Ct) as the discriminating factor, as this is highly dependent on factors that influence amplification efficiency; for example, DNA concentration, contamination, inhibitors and genetic variants which will inevitably characterise and vary in field samples. These variables will need to be investigated further using synthetic DNA template before examining field samples. Once these limitations can be overcome, the method could also be adapted to identify the frequencies of alleles conferring resistance to other anthelmintic drug groups once molecular markers are identified, and in other GIN species. This would have applications in informing sustainable GIN control in individual flocks or herds of ruminant livestock.

## Supplementary Information

Below is the link to the electronic supplementary material.

Supplementary material 1 (DOCX 15.6 kb)
