# Supplementary material for: A loop-mediated isothermal amplification (LAMP) assay to identify isotype 1 β-tubulin locus SNPs in synthetic double-stranded Haemonchus contortus DNA
Source: J Parasit Dis. 2021 Jul 5;46(1):47–55. doi: 10.1007/s12639-021-01414-w (PMC8901900; doi:10.1007/s12639-021-01414-w)
Supplement: Supplementary file 1 — Supplementary material 1 (DOCX 15.6 kb) [file 12639_2021_1414_MOESM1_ESM.docx]

**Supplementary material**

**S1.** Consensus sequences used to design of LAMP primers specific to *Haemonchus contortus*

>Teladorsagia_circumcincta/1-351 CON

GGAGGAGGWACTGGTTCGGGTATGGGCRCTTTGCTYAYCTCMAAARTTCGCGAKGAGTATCCGRRTAGARYYATGGCYTCATTCTYMGTTGTYCCATCRCCWAAGGTWDYKTATYYCTDRCRGYMGYCSTYTTTCRARATBGHRTKTACAGGTTYYHVARKDGCAAYCCATTTTMMGARCTCTYHVCAYYRATGCKYWAYHGTGRADTGTGCRRHGAWRTTRTRTTYWTAGGTWTCCGAYACYGYTGTGGARCCTTACAATGCCACTCTTTCTGTWCACCARTTGGTWGAAAAYACYGAYKWRACRTWCTGYATCRATAATRARGCTCTSYACGATATCYGCTTCCGCACA

>Trichostrongylus_colubriformis/1-317 CON

GGAGGAGGTACTGGAYCYGGTRTGGGMACTYYKCTWATYYCRAAARYYCGTGARGAGYRCCCDGAYAGARTTRTGGCTTCGTHCTCTGTYGTTYCWTCACCYAAGGTRCARWYABCYMYMRNHDBRKSSVWTAYCTTYYRYWRNYRTTTAWWCTYRSRCTRAAWTKKDTBAVHRATGCGWAWMAVRDVYWTTYMRAGGTTTCCGWCACYRYYGTGGARCCCTACARTGCTAYTYTDTCWGTMCAYCARCYGGTAGWRARYACCGRYGARRCWTWCTGYAHYGATAAYGRAGCTCYBYAYGRTRTYTKYTTCCGCMCA

>Oesophagostomum_columbianum/1-355 CON

RMYKYRKTSRMKYSGWRSARSKMMMSKYTYKSSKWTSRSMASYYKSYTSAKWWYAMAGATSMGWSWKKMWKWMYCRRMWAKGAWSMTKTSYWYRWTMTMTGYKKYMSCWYYACCTAAGGTCTCGGATACTGTGGTAGAGCCGTACAACGCCACGCTTTCCGTTCACCAGCTGGTTGAGAATACAGATGAGACCTTCTGTATCGACAATGAAGCTTTGTATGATATTTGCTTCCGCACTTCGGATACTGTGGTAGAGCCGTACAACGCCACGCTTTCCGTTCACCAGCTGGTTGAGAATACAGATGAGACCTTCTGTATCGACAATGAAGCTTTGTATGATATTTGCTTCCGCACT

>Haemonchus_contortus/1-348 CON

GGAGGWGGCACYGGATCTGGARTGGGCACTTTRTTAATTTCAAAAATTCGTGRAGAGTACCCTGATAGAATTATRGCTTCGTWCYCCGTTGTWYCATCACCCAAGGTGRGATCGTGTTRATCYTTBCTYTTTTCCTARATTGTGYATTTGAATTAYTTAYCCTCWTGARRAATYYAAGTYDWARTAAGYCYCRYCACCTGTAAACRTGHKAAAGGARRAYGTTYTAAGGTRTCCGACWCTGYYGTRGAMCCCTRCAATGCTWCCCTTTCCGTCCRTCAAYTGRTAGARAAYACCGATKARACATWCTGTATTKACAACGAAGCYCTGTATKATATCTGCTTCCGCACA

**S2.** LAMP primers that were tested, but not selected, to amplify the *Haemonchus contortus* F167Y isotype 1 β-tubulin SNP allele.

| **SNPs - Primer** | **Sequences 5' - 3'** |
| --- | --- |
| **Primer set 91** | |
| 167F3-91 | ATTCACTTGGAGGAGGCA |
| d167B3-91 | GTTHACAGGTGRTGRGACTT |
| S167FIP-91 | GAACGAAGCCATAATTCTATCAGGGCTGGAATGGGCACTTTGT |
| d167BIP-91 | CACCCAAGGTGAGATCGTGTNACTBRDATYYTBANGDGGAT |
| **Primer set 99** | |
| 167F3-99 | TCAAAAATTCGTGAAGAGTACC |
| 167B3-99 | ACGACAGTGTCGGATACC |
| S167FIP-99 | AAGCAAAGATTAACACGATCTCACCTGATAGAATTATGGCTTCGTTCT |
| 167BIP-99 | ATCCTCATGAAGATCCAAGTTGAAATTAAAACATCTTCCTTTCACATG |
| **Primer set S** | |
| 167 Seamus F3 | GCTGAAGGTTGTGATTGC |
| 167 Seamus B3 | CTTGGATCTTCATGAGGATAAG |
| 167 Seamus FIP | AGTGAATGCGTCAATTGGAAGCTTCAGGTACTGACTTCATCAAC |
| S167 Seamus BIP | AGGAGGCACTGGATCTGGAAACCTTGGGTGATGGAACAACGGAGAA |
| **Primer set 76** |  |
| S167F3-76 | TCAAAAATTCGTGAAGAGTACC |
| S167B3-76 | ACGACAGTGTCGGATACC |
| S167FIP-76 | GCAAAGATTAACACGATCTCACCTTTGATAGAATTATGGCTTCGTTCT |
| S167BIP-76 | ATCCTCATGAAGATCCAAGTTGAAATTAAAACATCTTCCTTTCACATG |

The underlined nucleotides show the SNP. The primers were degenerate using IUPAC code. Y: C/T; K: G/T; R: A/G; D: A/G/T; H: A/C/T; V: A/C/G; B: C/G/T; N: A/C/G/T. All of the synthetised primers were HPLC grade (Integrated DNA Technologies, Leuven, Belgium).
